# Prevention of antibiotic resistance – an epidemiological scoping review to identify research categories and knowledge gaps

**DOI:** 10.1080/16549716.2020.1756191

**Published:** 2020-06-01

**Authors:** Stig Wall

**Affiliations:** Department of Epidemiology and Global Health, Umeå University, Umeå, Sweden

**Keywords:** Antimicrobial Resistance, Antibiotic resistance, antimicrobial resistance, drug resistance, prevention, health policy, behaviour, global threat

## Abstract

**Background:** Antibiotics have become the cornerstone for the treatment of infectious diseases and contributed significantly to the dramatic global health development during the last 70 years. Millions of people now survive what were previously life-threatening infections. But antibiotics are finite resources and misuse has led to antibiotic resistance and reduced efficacy within just a few years of introduction of each new antibiotic. The World Health Organization rates antibiotic resistance as a ‘global security threat’ impacting on global health, food security and development and as important as terrorism and climate change.

**Objectives:** This paper explores, through a scoping review of the literature published during the past 20 years, the magnitude of peer-reviewed and grey literature that addresses antibiotic resistance and specifically the extent to which “prevention” has been at the core. The ultimate aim is to identify know-do gaps and strategies to prevent ABR.

**Methods:** The review covers four main data bases, Web of Science, Medline, Scopus and Ebsco searched for 2000–17. The broader research field “antibiotic OR antimicrobial resistance” gave 431,335 hits. Narrowing the search criteria to “Prevention of antibiotic OR antimicrobial resistance” resulted in 1062 remaining titles. Of these, 622 were unique titles. After screening of the 622 titles for relevance, 420 abstracts were read, and of these 282 papers were read in full. An additional 53 references were identified from these papers, and 64 published during 2018 and 2019 were also included. The final scoping review database thus consisted of 399 papers.

**Results:** A thematic structure emerged when categorizing articles in different subject areas, serving as a proxy for interest expressed from the research community. The research area has been an evolving one with about half of the 399 papers published during the past four years of the study period. Epidemiological modelling needs strengthening and there is a need for more and better surveillance systems, especially in lower- and middle-income countries. There is a wealth of information on the local and national uses and misuses of antibiotics. Educational and stewardship programmes basically lack evidence. Several studies address knowledge of the public and prescribers. The lessons for policy are conveyed in many alarming reports from national and international organizations.

**Conclusions:** Descriptive rather than theoretical ambitions have characterized the literature. If we want to better understand and explain the antibiotic situation from a behavioural perspective, the required approaches are lacking. A framework for an epidemiological causal web behind ABR is suggested and may serve to identify entry points for potential interventions.

## Background – the emergence of a global health problem

Antibiotics have become the cornerstone for the treatment of infectious diseases and contributed significantly to the dramatic global health development during the last 70 years. Millions of people now survive what were previously life-threatening infections. But antibiotics are finite resources. Misuse has led to antibiotic resistance (ABR) and reduced efficacy within just a few years of introduction of each new antibiotic [1]. The problem was foreseen by Alexander Fleming in his Nobel speech in 1945 when he concluded that ´*the time may come when penicillin can be bought by anyone in the shops. Then there is the danger that the ignorant man may easily underdose himself and by exposing his microbes to non-lethal quantities of the drug make them resistant´*. But, as late as in 1968, the US Surgeon General William H Stewart stated that ´*it is time to close the book on infectious diseases and declare the war against pestilence won´* [2].

The development of ABR is a direct response to the misuse of antibiotics in healthcare, the animal industry, agriculture and aquaculture. Misuse refers to use without need, use without prescription, self-medication, irregular or interrupted dosing or sharing of antibiotic. Combating ABR by detecting, preventing, and controlling resistance requires strategic, coordinated, and sustained efforts. This calls for national and international engagement of governments, academia, industry, healthcare providers and the general public. This will not only support patient care but also foster economic growth and national security [3].

Antimicrobial resistance (AMR) is a broader term, encompassing resistance to drugs to treat infections caused by bacteria and other microbes such as parasites (e.g. malaria), viruses (e.g. HIV) and fungi (e.g. candida). Incentives for the pharmaceutical industry to develop new drugs are counteracted by our ambition to limit antibiotic use and industry priorities are therefore often directed towards investment in medicines to treat chronic diseases that require long-term consumption. Even if new successful antibiotics are introduced, international coordination and commitment is urgently needed to establish a ‘one health’ strategy to address regulation, independent monitoring and policing [4–8].

Behind the development of resistance are also knowledge gaps, preconceived notions, lack of adherence in patients to prescribing and in prescribers to guidelines.We need to raise awareness about ABR, change behaviours, develop context-specific guidelines for prescribers, and introduce regulations for use in all areas. Industry needs to work with policy-makers and embrace new business models to prevent the spread of antibiotic-resistant infections [9].

Every year, 700,000 people die from resistant infections. In their final report, the Wellcome Trust and the British Government estimated that, at the current rate of increase, 10 million people will die during the year 2050 as a result of AMR [10]. Ten million deaths in 2050 would exceed the 8.2 million cancer deaths in 2019 [11].

According to the World Health Organization (WHO) antibiotic resistance is a ‘global security threat’ impacting on global health, food security and development and as important as terrorism and climate change. The World Bank estimates that the economic impact may be greater than from the 2008–09 global financial crisis, cutting between 1.1 and 3.8 percent of global gross domestic product but not just felt in the short term as was the case during the recession [12]. The true cost of not being able to use antibiotics routinely is difficult to calculate [13]. Cost estimates have been crude, being current rather than future and applying to developed, rather than developing countries [14].

Antibiotic resistance can affect people of all ages in all countries. It is estimated that 5.7 million people, most in low- and middle-income countries (LMICs), die each year from treatable infectious diseases. Many lives could have been saved if antibiotics had worked and been available. This number is far more than the 700,000 annual deaths worldwide that are due to ABR. The development of resistance, while threatening our enforced right to best care, reminds us that the world’s poorest are still more affected by lack of access to antibiotics than by the resistance. Preserving antibiotic effectiveness while ensuring universal access, is thus an ethical obligation [15].

Antibiotics are a common global utility with limited sustainability which has been overlooked in the implementation of the Sustainable Development Goals (SDGs) and should be acknowledged along with other fundamental global concerns, such as climate change. This may help us to understand the consequences of not taking action [16].

The loss of efficacy of antibiotics through overuse and misuse has been labelled a ‘tragedy of the commons’ which occurs “when people in a community squander a limited, shared resource, as each actor pursues her own short-term self interest by exploiting the resource for private benefit” [12].

This paper explores, through a scoping review of the literature published during the past 20 years, the magnitude of peer-reviewed and grey literature that addresses ABR or AMR and specifically the extent to which “prevention” has been at the core. It is not a systematic review of research questions and does not follow the standard format for such. The ultimate aim is to identify know-do gaps in research and strategies to prevent ABR.

The report does not claim to cover research at laboratory or preclinical levels but rather takes a population-based epidemiological and behavioural perspective on the topic “prevention of antibiotic resistance”. Although the literature often addresses the broader field of antimicrobial resistance, this report, in general, deals with antibiotic resistance.

## Method – searching for literature on “prevention of antibiotic resistance”

During 2018, a structured search in the published literature (2000–17) was conducted across four main data bases, Web of Science, Medline, Scopus and Ebsco. A “scoping review” aims ‘to synthesize research evidence and to categorize or group existing literature in a given field in terms of its nature, features, and volume’. Specifically, the aim was to arrive at a thematic overview of the research area “prevention of antibiotic resistance” and ultimately to identify relevant research categories and knowledge gaps.

**Figure 1.**
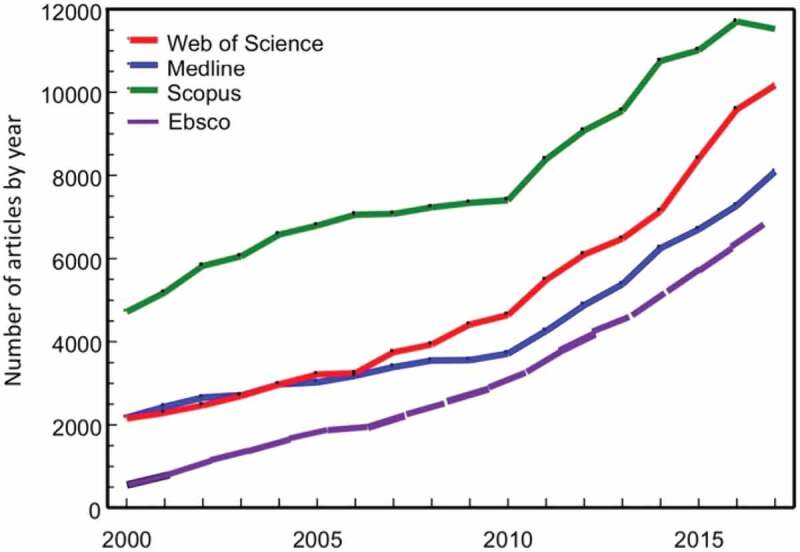
Time trends of published articles 2000–17 matching the search criterion ‘antibiotic OR antimicrobial resistance’ in four library databases

Figure 1 shows that the broader research field has been increasingly addressed during these 18 years, and the search for “antibiotic OR antimicrobial resistance” gave 431,335 hits with a three-fold increase between 2000 and 2017. These are not unique hits since there are overlaps between the data bases, especially between Web of Science and Medline.

In the search for “prevention” among these articles, proximity rules were used by requiring some closeness between “prevention” and “antibiotic OR antimicrobial resistance” in titles or abstracts. “Prevention” or linguistic inflections thereof should be no further away than three words for the paper to be included. Technically this is specified as “prevent* N3 antibiotic resistance OR prevent* N3 antimicrobial resistance” in the search.

**Figure 2.**
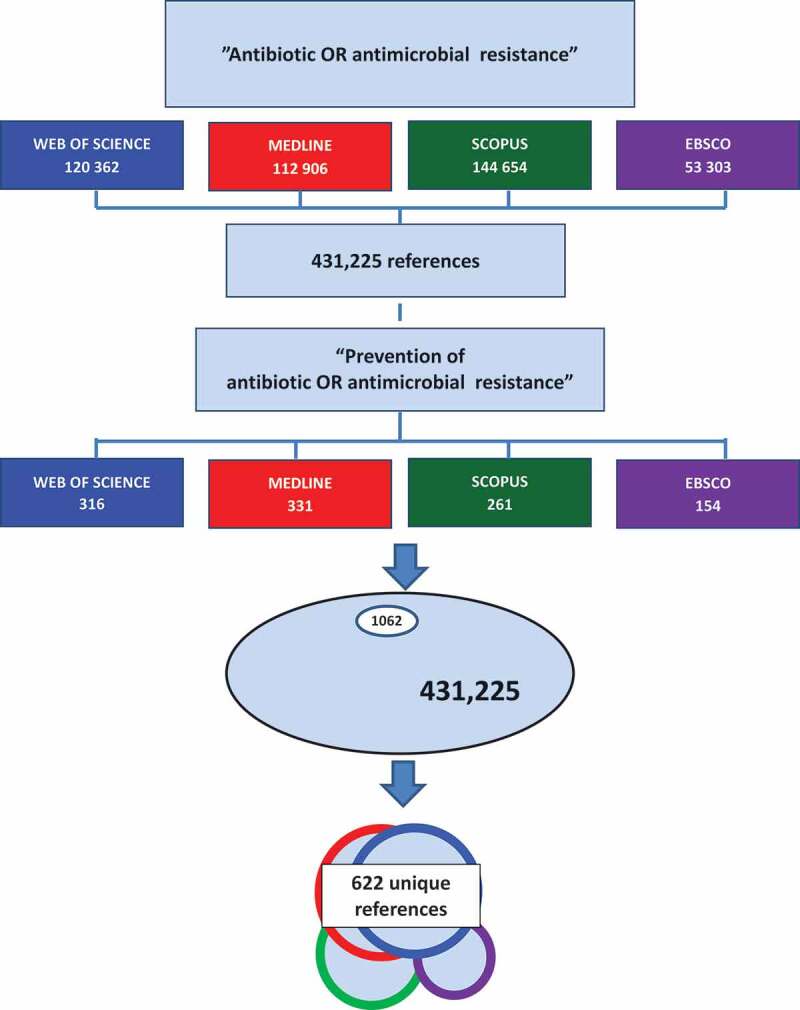
Flow chart for the selection of papers on the prevention of antibiotic or antimicrobial resistance resulting in 622 unique references

Matching for each of the data bases to the narrowing of the search criteria resulted in 1062 papers remaining of the initial 431,335, i.e 0.25% (Figure 2). Of these, 622 were unique titles.

After screening of the 622 titles of these references for relevance, 420 abstracts were read and screened for relevance and of these 282 papers were included and read in full. An additional 53 references were identified from these papers, and 64 published during 2018 or 2019 were also included. The final scoping review database thus consisted of 399 papers.

## A thematic structure

The research area has been an evolving one with half of the papers published during the past four years of the study period. A large proportion of papers addressed how ABR can be counteracted within health facilities through better compliance with treatment guidelines and also through national and global strategies. Epidemiological surveillance methods provide valid tools for this. The historical background referring to the relatively short periods during which antibiotics have been used and resistance has evolved, has sometimes been used as the basis for a number of scenarios modelling the impact of no action. The ABR field is increasingly recognized as a national and international policy issue. A number of papers emphasise the importance of ABR being dealt with as a health system failure arguing that the perspective should be that of “one health” cutting across human health, animal industry and the environment. Alternatives to antibiotics are on the agenda, mostly through pre- and probiotics. Guidelines and strategies are often implemented as stewardship or educational programmes, while others address knowledge or awareness among the public or the prescribers. Considering the importance of preventing infections in the first place, it is noted that, proportionately, few papers are dealing with mechanisms or how to enhance the immune system.

**Figure 3.**
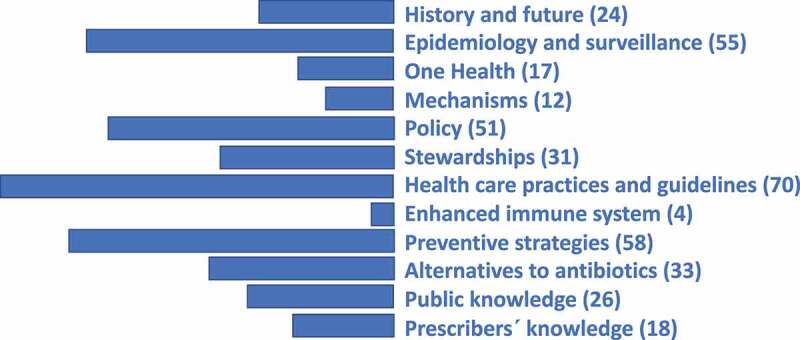
A suggested thematic structure of the research area “prevention of antibiotic or antimicrobial resistance” based on the 399 articles remaining after literature search and screening for relevance

While reading the papers, each paper was classified by the author reflecting its main scope. A thematic structure of possibly overlapping subject areas thus gradually emerged. Figure 3 may thus serve as a proxy for interest expressed from the research community relating to prevention potentials during the past 20 years.

**Figure 4.**
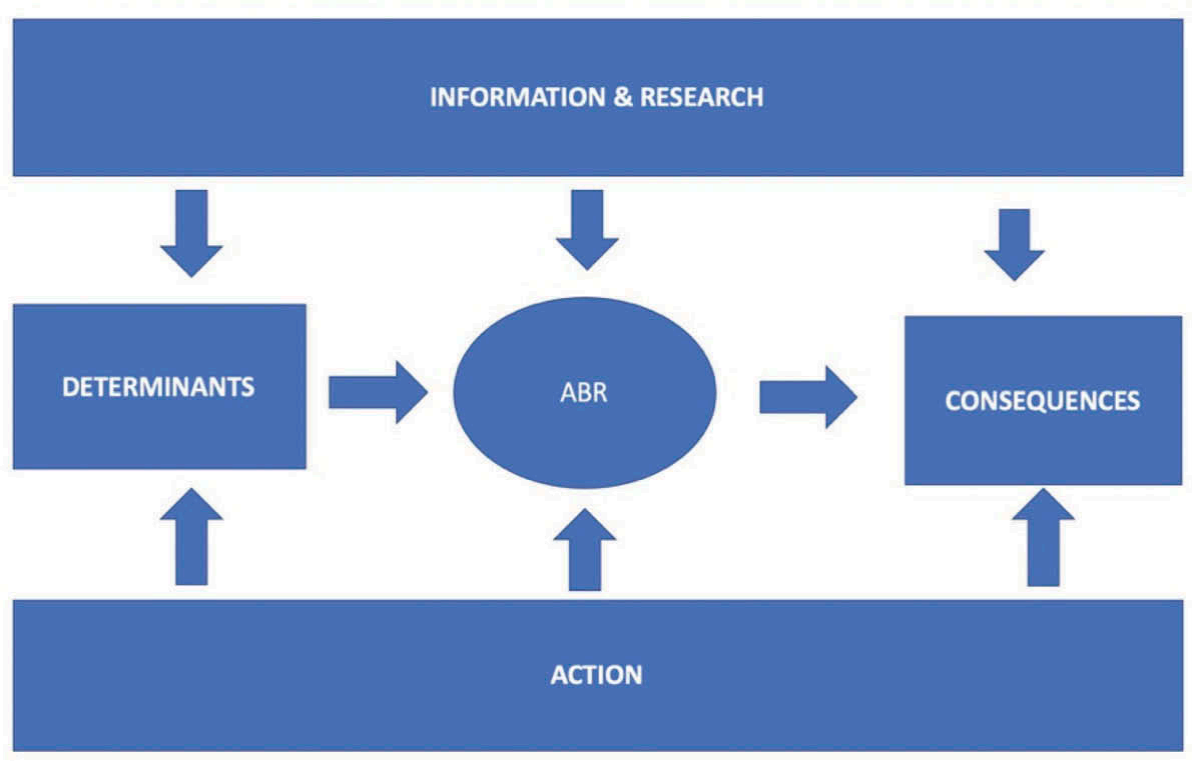
An epidemiological framework of the research area “prevention of antibiotic or antimicrobial resistance” suggesting a causal web towards ABR and its consequences

Figure 4 provides an initial framework for an epidemiological causal web behind ABR and which will be pursued in the assessment of the literature following the themes in Figure 3. Socio-cultural and individual determinants along with biological characteristics are driving ABR occurrence and variation. These background factors are influenced by the levels and types of action from communities and administrative bodies. In parallel, knowledge development is on-going generated by official sources, bio-medicine and health care with input from research. The consequences in terms of individual and population health are all dependent on ABR development modified by the outcome of new knowledge and the impact of prevention, infection control and resistance mitigation.

## Epidemiology and surveillance

Epidemiological data provide knowledge about the distribution of factors that determine health and disease in populations. Data on the epidemiology of ABR and infectious diseases are essential to inform our understanding of the patterns of use and misuse of antibiotics and their consequences [17].

Diarrhoeal diseases impose a substantial burden on LMICs, being the second most common cause of death amongst children, and claiming 1.1 million lives each year. Sixty percent of these deaths are associated with inadequate access to safe water and sanitation. It is estimated that improved sanitation infrastructure in India will result in 590 million fewer diarrhoea cases being treated with antibiotics by 2030 [18]. Epidemiological predictions of the mortality consequences of AMR show that 90% of the global impact in the next 30 years will be in Africa and Asia [11] where the high incidence of diarrhoeal diseases is largely due to the lack of universal access to safe water and sanitation.

Even though seventy percent of diarrhoeal episodes are viral rather than bacterial, antibiotics are often first-line treatment. Modelling suggests that across four middle-income countries (India, Indonesia, Nigeria and Brazil) close to 500 million courses of antibiotics are used to treat both bacterial and viral diarrhoea each year. It is estimated that diarrhoeal disease in these countries would be reduced by some 60 percent with universal access to improved water and sanitation [10]. Nevertheless inappropriate drug use for diarrhoeal disease is a major cause of increasing ABR [19].

The global divide is illustrated in many contrasting international scenarios. While many children in high-income countries (HICs) are prescribed antibiotics for common upper respiratory infections, death tolls are high in LMICs because antibiotics do not work. In India, 58,000 infants died in 2013 from resistant bacterial infections [20]. Overconsumption in the developed world is illustrated by the fact that infants and toddlers spend a mean of 42 days on antibiotics in the first year of life, a mean of 49 days on antibiotics in the second year of life [21] and that 85% of children aged under two years who are diagnosed with acute otitis media, are prescribed antibiotics for this condition [22].

The epidemiology of ABR also includes factors beyond individual control and that require interventions at national levels. These factors vary by regions and global action requires combined international efforts [23]. A study among 27 European countries concluded that a nation´s educational level and governance system impacted on antibiotic use with higher consumption in more poorly managed countries in which national educational standards were lower [24]. Specifically, countries with high levels of corruption had higher antibiotic consumption rates, suggesting that ABR is not solely a medical, but also a behavioural and a social problem. These findings were corroborated in a multivariate analysis of data from the European Quality of Government Index, in which corruption accounted for a significant amount of the between-region variation in antibiotic consumption. Even after controlling for socio-economic development, dysfunctional public institutions in the health sector were responsible for much of the inappropriate antibiotic consumption [25].

In a global assessment for 103 countries, after accounting for type of governance, education, economy, health care spending and community infrastructure, it was concluded that reducing antibiotic consumption alone would not control resistance. Independent of antibiotic consumption, poor infrastructure (e.g. sanitation), poor governance (e.g. corruption) and low health expenditure were all associated with higher rates of resistance [26].

Another study in 17 European countries concluded that socio-economic factors could help explain variation in outpatient antibiotic use. The authors emphasised that supply-side factors also play a role. More doctors and a fee-for-service remuneration principle were associated with higher consumption of antibiotics [27] and a 1% increase in doctor-to-population density has been associated with a 0.52% to 0.86% increase in outpatient use of antibiotics [28].

The positive impact of higher education on appropriate antibiotic use does not always hold. In China, which represents one fifth of the global population, misuse of antibiotics, including self-medication and buying over-the-counter without prescriptions, was higher among young well-educated university students [29]. It has been estimated that each Chinese resident takes 138 g of antibiotics per year, tenfold greater than the average in the US. The unnecessary use of antibiotics for pediatric outpatients with upper respiratory infections was also higher in township hospitals than in county hospitals and village clinics [30]. A study in Nigeria reported that three out of four under-five children were given un-prescribed antibiotics for upper respiratory infections with this being more common among more educated mothers [31].

Access to antibiotics is critical in maternity units. Preventable maternal or neonatal mortality is still unacceptably high in LMICs where many women do not have access to skilled health care and hygienic birthing facilities. However, we lack information on antibiotic use in these settings [32].

When explaining variation in antibiotic usage, qualitative research and the social sciences can help us to understand the effects of underlying behavioural, social and cultural norms and practices [33]. For example, hierarchical societies use more antibiotics than egalitarian countries. Health care financing and reimbursement systems influence the way in which social and economic factors determine health. Contextual factors refer to how patient care is differently organized and this can indirectly influence prescriptions and guidelines. Behavioural aspects are exemplified by physicians´ knowledge and attitudes towards diagnostic uncertainty and treatment principles. Yet, making changes is challenging and demanding for the patient-physician in building trust [30,34]. Doctors may need to be convinced that communication rather than prescription satisfies the patient [35].

In an attempt to explain the wide differences in the prevalence of antibiotic-resistant pneumococci between France (43%) and Germany (7%) based on clinical isolates of *S. pneumoniae* collected from patients of all ages throughout Europe, the authors argued that the cultural and socioeconomic environment contribute substantially to prescribing practices and that failure to understand these perspectives may disable intervention ambitions [36].

Surveillance programmes are crucial for understanding and control of resistance. Valid surveillance can be incorporated into longitudinal cross-country studies to follow the changes in resistance over time in different areas [37]. The European Center for Disease Prevention and Control (ECDC) first reported AMR surveillance in Europe in its 2014 Report [38]. A population modelling analysis using ECDC data estimated the burden of antibiotic resistance in 2015 using disability-adjusted life years (DALYs). Out of 671,689 antibiotic-resistant bacteria infections, two thirds were associated with health care, accounting for an estimated 33,110 attributable deaths and 874,541 DALYs. The overall DALY rate, 170 per 100,000 population, is similar to the combined burden of HIV, influenza, and tuberculosis in the European Union (EU). The burden was highest in infants and people aged 65 years or older, and overall in Italy and Greece [39,40].

Modelling, e.g. the one predicting 10 million deaths due to AMR in 2050 [10], has been criticized for not using information from the clinical institutions where most deaths from resistance take place. Critics have also claimed that global estimates are unreliable and hence undermine rather than support the fight against AMR in a post-antibiotic era. The major shortcomings in global estimates, however, are the lack of reliable population-based data from LMICs [41].

In 2015 WHO launched the Global AMR Surveillance System (GLASS). Forty-two countries enrolled in a standardized approach to share global data on AMR, inform decision-making and drive advocacy and action on various levels [37]. GLASS represents a major shift from surveillance approaches based solely on laboratory data to a system that includes epidemiological, clinical and population-based data [42,43]. This is also a prerequisite for the much needed bridging across bench- and bed-based research [44].

In 1997, the Swedish Medical Journal reported a high frequency of ABR in Swedish intensive care units and it was recommended that resistance status should be monitored continuously in order to prevent suboptimal antibiotic use [45]. Today, more than 20 years later, Sweden has the lowest antibiotic use in the EU. The decline in use since 1992 has been 51 per cent and antibiotic use in Sweden is now below 300 prescriptions per 1,000 inhabitants [46]. This is, to a large extent, due to STRAMA, the national strategic program against ABR. This initiative started as an informal network between experts and authorities, later led to a national action plan and eventually in 2006 to a government bill [47]. STRAMA has since worked across sectors and disciplines to promote the rational use of antibiotics by providing feedback to clinics with local data and treatment guidelines. Despite this, there is also an increase in resistance in Sweden and the number of reported cases in 2018 was, according to the Public Health Agency, 15,000. With the current rate of development, this may amount to 71,000 cases in the year 2050. A recently published microsimulation analysis suggests that, if trends continue, the current costs will have doubled by 2030 and increased four-fold by 2050. The total future cost implication for health care until 2050 is estimated at 16 billion SEK notwithstanding indirect social costs [48,49].

## One health – an interdisciplinary and intersectoral issue

The One Health approach has been defined as ‘ … the collaborative effort of multiple disciplines – working locally, nationally and globally – to attain optimal health for people, animals and our environment … ’ [8]. Health of people, animals and the environment are connected and ABR is acquired not only through human use but can be transferred to humans through selective pressure in the environment. Antibiotic residues in soil, waste water and manure also have a greater impact for resistance than previously thought [5]. Also, while antibiotics as growth promoters in animal production are banned in Europe, without any documented side effects on food availability, quality or price [50], their use for this purpose is extensive in LMICs.

One Health requires research, surveillance, and interventions across sectors and there is a gap to be bridged between the biomedical, social, environmental and animal sciences [51]. There is a lack of implementation research that tap into the wealth of knowledge that already exists [52]. But some positive efforts are being made. A study in the Netherlands demonstrated a 47% drop in the use of antibiotics in the dairy sector between 2009 and 2015. This example showcases how behaviour change can be achieved with cooperation between stakeholders [53].

There is consensus that the interplay between sectors impacts on ABR locally and nationally, as well as globally. Animals raised for food use the majority of antimicrobials sold globally and some studies have documented antimicrobial use in animal production in LMICs [54]. One recent example comes from Pakistan where there has been indiscriminate use in the broiler industry with annual figures as high as 560 tons [55]. In a recent global mapping through 901 point prevalence surveys, the largest ABR hotspots were in China and India. Between 2000 and 2018, bacterial strains showing resistance above 50% increased from 0.15 to 0.41 in chickens and from 0.13 to 0.34 in pigs [56].

An international workshop, which included scientists from 14 countries, was held in Gothenburg in 2017. Participants stressed the need to evaluate and develop social interventions, to better understand the role of anthropogenic processes in the environment leading to resistance, and the need for quantitative risk assessment of the impacts of exposure via environmental routes on human and animal health. Putting a price tag on ‘business as usual’ might motivate actions and, in quantifying the economic burden of non-action, lessons might again be learned from the climate challenge [57].

The WHO Global Action Plan on AMR [58], emphasised the need to also address economic forces behind infectious diseases and resistance development. While low-income countries are burdened by disease-causing environments as well as limited access to antibiotics, farmers lacking veterinary services turn to antibiotics. This further illustrates that contextual and cultural determinants need to be tackled in global endeavours. It has also been suggested that, by recognizing the analogies with other global concerns such as climate change, we will better understand the irreversible consequences resulting from not taking action [16].

The One Health Strategy requires nations to implement surveillance and report human, animal and environmental data jointly [7]. There is still a lack of data from LMICs on antibiotic resistance as a cause of death. A major improvement would be to add this as an important study area for the many well functioning health and demographic surveillance sites (HDSSs) in Africa and Asia mastered through the INDEPTH Network (www.indepth-network.org). In an on-going study, this infrastructure will be used to assess the access, provision and use of antibiotics in three African and three Asian countries [59].

In an international workshop organized by the US Academy of Sciences in 2017 it was acknowledged that AMR needs to become a high-profile social issue, like cancer, tobacco control and HIV/AIDS, to provide the necessary stimulus to drive change and be of relevance to each and everyone. What may be regarded as a distant and abstract issue might then be made more personal and humanized. Yet more people die from lack of access to antimicrobials than from resistance. This is why dealing with the tension between lack of access and excess use is critical [6].

Although several political declarations call for coordinated global action in adopting the One Health principles, it is disappointing that antibiotic resistance is not more visible in the UN SDGs since achieving them requires antibiotic resistance to be addressed [60,61] while progress on the SDGs will in fact contribute to tackling AMR [52]. The Interagency group (IACG) between FAO (Food and Agricultural Organization of the United Nations), OIE (World Organization for Animal health) and WHO, resulting from the UN Report “No time to wait – securing the future from drug-resistant infections” [62] was given terms of reference to implement the WHO Global Action Plan on AMR in support of the SDGs while also regularly reporting on progress. The solution, as for climate change, needs global action whereby nations work together for the common good.

## Understanding the drivers behind antibiotic resistance

At the cellular level ABR genes (DNA) can be transferred between bacteria either vertically or horizontally. Vertical spread means that a bacterium mutates and its DNA is inherited from parent to daughter cells when a bacterium divides. Horizontal spread means that the DNA is transferred between bacteria of the same or different species in their micro-environment. There are three different mechanisms by which this occurs – *conjugation* through transfer of plasmids between bacteria, *transformation* through uptake of DNA from dead bacteria in the environment, and *transduction* where genes are transferred between bacteria through a bacteriophage, a virus infecting the bacteria [63].

To assess the impact of different factors at the population level, a two-round Delphi study was undertaken of the relative contribution to ABR of e.g. human and animal use, environmental contamination, health care transmission, diagnostics and dosing, travels and drug administration. The evidence was identified through a review of the literature using the GRADE criteria to rate the quality of the evidence [64].

The results of the study showed that human and animal misuse/overuse of antibiotics were the highest contributors to resistance and they also had the greatest population impact. The contribution from health care transmission of antimicrobial-resistant microbes was almost as high. The population impact from travel, mass drug administration and vaccination was relatively lower. More evidence was requested to assess the potentially important contribution from treatment guidelines on one hand and environmental contamination on the other [64].

Any reversal of ABR is complex and varies with antimicrobial consumption patterns, bacterial species, lifestyle, and the environment [65,66]. More attention needs to be focused on patient expectations and perceptions of illness and the constraints of medical practice. We also need to better communicate with individual patients and inform the general public about the risks of inappropriate antibiotic use.

A key aspect of this concerns the prescribed treatment dose [67]. The importance of proper dosage has long been urged as a means of preventing the emergence of resistance [68]. Understanding these mechanisms is important both for designing research studies and for treatment protocols [69]. We lack epidemiological data on changes in resistance following decreased drug consumption [65]. Study designs to assess the direct effects of antimicrobial use on the development of resistance will need clinical studies and include data on bacterial culture and colonization. The assessment of treatment effects may involve time-to-event models controlling for duration and dose. Effect modification may be assessed by controlling for exposure to other patients carrying resistant bacteria. For interpretation of intervention studies we need randomized studies with proper control groups.

For prescribers, knowledge about mechanisms matter for making well-informed decisions about the choice of antibiotics and dosage. For policymakers, they matter when designing campaigns for encouraging rational regulated antibiotic use. Decision-makers also need knowledge on the determinants of the variation in antibiotic use and resistance patterns [34].

## Antimicrobial stewardship – a safeguard for the future?

Antimicrobial stewardship aims to promote the responsible use of antimicrobials, improve patient outcomes, reduce the emergence of AMR and decrease the spread of infections caused by multidrug resistant organisms [70]. Responsible use should balance the individual´s right to treatment with society´s longer term need for sustained access to effective therapy [71]. The means to achieve this involves the implementation of policies which include evidence-based guidelines for prescriptions [72]. Although it has been suggested that stewardship programmes can reduce antibiotic consumption and resistance, adherence to such programmes is not straightforward in LMICs. Poor countries lack surveillance and adequate infection control and the prohibition of over-the-counter sales of antibiotics is not easily implemented when prescribers are lacking [70].

A recent systematic review of eight stewardship programmes found no solid evidence of their effectiveness in reducing antibiotic resistance in hospital settings. This is attributed mainly to suboptimal study designs and heterogeneous program features, preventing the pooling of studies [72].

Another systematic review of the effectiveness of 27 stewardship programmes in hospitals in two low- and 11 middle-income countries showed positive effects regarding the use of antibiotics in most studies. However, it was difficult to draw firm conclusions because of heterogeneity across programmes and weak study designs [73]. Specifically, it was concluded that, since stewardship programs in hospital settings aim at changing prescribers´ behaviour, determinants of such should be included in the programs involving also behavioural scientists.

In order to better understand the barriers and enablers for implementing antimicrobial stewardship, a qualitative study was undertaken in Australian regional and rural hospitals [74]. The key barriers were lack of education and specialist support while occupational pride and a flat governance structure were perceived as major enablers. This is consistent with the twenty year experience of the Swedish STRAMA group where continuous dialogue with regional and local clinics, with audit and feedback recommendations based on local data, has been instrumental in achieving positive outcomes [47]. Similar experiences from the Netherlands suggest that effective surveillance and feedback by stewardship teams leads to more appropriate prescriptions, fewer infections, shorter hospital stays and less resistance [75]. One example of the importance of policy support is the Transatlantic Task Force on Antimicrobial Resistance established by the US and EU leaders in 2009 [76].

Education is a key element of stewardship and there are ample indications of knowledge gaps, for example among medical students in choosing therapy. A systematic review of 57 studies revealed poor knowledge among clinicians about the correct prevalence of resistance. They acknowledged the importance of antibiotic resistance but ‘not in their backyard’ [77].

However, education alone is not sufficient. It has to be contextualized and behavioural factors must be addressed in stewardship programmes. There are also cultural and behavioural norms around prescribing. Resistance to changing practice and the fear of losing autonomy is real. Evaluations of antimicrobial stewardship programmes therefore need social science and qualitative research methods rather than randomised clinical trials [78].

Stewardship programmes may also help prevent the spread of infections caused by damages to normal bacterial flora from inappropriate antibiotic use, which for instance can lead to *Clostridium difficile*. This has been linked to 14,000 deaths per year in the US [79]. A successful stewardship programme in US hospital settings documented decreased rates of *C.difficile* from 1.5 to 0.5 cases/1000 bed days over a two-year period [79]. A systematic review and meta-analysis of 32 studies, involving nine million patient-days, showed reductions in infection and colonization rates with antibiotic resistant bacteria and *C.difficile* up to 50% and which was improved further if co-implemented with hand-hygiene measures [80].

Most stewardship programmes have been designed for adults and fewer examples refer to pediatrics. Sweden can again demonstrate a good example where, in outpatient settings during 1992–2014, the total consumption of antibiotics decreased by 41%. In the under-five age group sales decreased by 76% from 1,328 to 320 prescriptions per 1,000 [81].

Outside health care, antimicrobial stewardship has mainly taken the form of community activities and campaigns. One such example is the European Awareness Day which has been held on 18 November each year since 2006 when it was launched by the European Centre for Disease Prevention and Control [82]. Another is the Global Hand Hygiene Day launched on 5 May 2017 with the message ‘Fight antibiotic resistance – it´s in your hands!’ [83].

While evidence on the effectiveness of antimicrobial stewardship programmes basically comes from hospital settings, there is less evidence from communities and public campaigns. Also, most studies have been performed in high-income settings in Europe, the US and Australia. Antibiotic stewardship in LMICs, as it is included in the WHO Global Action Plan, not only addresses antibiotic use but also universal access to antibiotics when needed. Antibiotic stewardship programmes in LMICs are challenged by shortages of diagnostic facilities, clinical guidelines and unregulated prescriptions. Challenges are therefore special in resource limited settings and the causal link from consumption to resistance is less clear-cut than in high-income settings [84].

Antimicrobial stewardship has been put forward as a means of prescribing social norms [85]. However, disclosing the connection between individual behaviour and population resistance is a major challenge. There is a need to make the social cost more visible. According to the UK National Institute for Health and Care Excellence (NICE), nine out of ten general practitioners (GPs) say that they feel pressured to prescribe antibiotics and 97% of patients who ask for antibiotics are prescribed them [86]. An example of how feedback can change prescribing behaviour was shown in a pragmatic randomized controlled trial targeting 1581 top GP prescribers in the UK. The first group of GPs was given information about how their high prescribing rates deviated from the norm and the second group was exposed to an education campaign about prescribing. The first group had significantly fewer prescriptions at follow-up [87] than the second group. This again illustrates how behavioural processes, here the human wish to join the group and feel included, may impact on prescribing practices [85].

## Antibiotic resistance – a policy and health systems issue

Antibiotic resistance has since long been high on the agenda of international organizations like WHO [88] and features in national strategic plans. The WHO role is to stimulate political commitment, advocate action, initiate collaborations and facilitate the development of guidelines. The WHO support for global surveillance strategies is critical [89].

The authors of a policy analysis comparing England, France and Germany, concluded that, in order to address ABR, partnerships between health care professionals and patients are needed as is democratized decision-making and a balance between regulation and persuasion [90].

The absence of progress in combating ABR is partly due to a poor evidence base for the effectiveness of numerous policies across the human health and animal sectors in both HICs and LMICs [91]. The basic premises of the One Health Strategy include universal access to antimicrobials and valid surveillance to monitor progress. Financing models need to separate research and development investments from revenue returns and market sales, while ensuring that those in need have access to affordable and effective drugs [9,92,93].

The May 2015 World Health Assembly adopted a Global Action Plan on AMR [58], which presented the following five objectives:

- to improve awareness and understanding of AMR through effective communication, education and training;
- to strengthen the knowledge and evidence base through surveillance and research;
- to reduce the incidence of infection through effective sanitation, hygiene and infection prevention measures;
- to optimize the use of antimicrobial medicines in human and animal health;
- to develop the economic case for sustainable investment that takes account of the needs of all countries, and
- to increase investment in new medicines, diagnostic tools, vaccines and other interventions.

The Lancet Commission on Infectious Diseases in 2013 called for data on the magnitude of the burden of AMR and its contributions to excess mortality to convince policy makers to act [93] and the message was heard: Three years later the final report from the Wellcome Trust and the British Government [10,11] conveyed the alarming scenario now often echoed, that, at the present rate of resistance development, 10 million people will die in 2050 due to AMR. These data, based on modelling have, as mentioned earlier, been challenged for unreliability and for undermining the work against AMR [41].

Another, more recent policy document is the report ‘No time to wait – securing the future from drug-resistant infections’ which was published by the UN Intergovernmental Coordination Group (IACG) led by the WHO Director-General [62]. The IACG addressed the Member States with five groups of recommendations urging the Members to: speed up the development and evaluation of new and alternative antibiotics; ensure equitable access to quality assured diagnostics; immediately stop the use of antibiotics in the animal industry, and strengthen development and research in the long-term. The process will be supported by an independent UN panel to monitor and provide regular reports to Member States on current knowledge and best evidence regarding AMR.

The Swedish work against ABR is led by the National Public Health Authority [94–97] and characterized by local and national interactions and cooperation. Sweden leads the independent organisation ReAct, (www.reactgroup.org), created in 2005 as an international network to advocate and stimulate global engagement and collaboration between organizations, individuals and stakeholders.

Policy frameworks have been called for based on local situational analyses to meet the needs of individual member states [98]. This was stressed in one of the very first policy documents from WHO on AMR [99]. Table 1 gives some examples of policy statements and recommendations issued over the past two decades. Following the way the issue has been conveyed as a public health problem over time, it is obvious that the statements have become increasingly alarming while also being compared to other global health problems, especially climate change. Here several similarities, such as the lasting impacts across generations, have been noted. In recent documents, the need to make AMR prevention a United Nations Sustainable Development Goal has also been emphasised [60].

**Table 1.** Examples of statements and recommendations in policy frameworks during the past 20 years

Organization	Statement	Recommendations	Year
WHO Global Strategy [99]	*”Antimicrobial resistance is a global problem that needs urgent action”.*	Reducing the disease burden and the spread of infection. Improving access to appropriate antimicrobials. Improving use of antimicrobials. Strengthening health systems and their surveillance capabilities. Enforcing regulations and legislation. Encouraging the development of appropriate new drugs and vaccines.	2001
US Academy of Sciences [100]	*”What is perhaps most notable today is the increasing degree to which microbial resistance has become an important health threat”.*	Implementing and expanding surveillance efforts. Expanding professional education and training. Conducting economic studies to complement epidemiological studies.	2003
WHO Policy Perspectives on Medicines [88]	*”Antimicrobial resistance is one of the world’s most serious public health problems”.*	Mandated multidisciplinary national task force to coordinate policies and strategies to contain AMR. National reference microbiology laboratory coordinating a network of reliable diagnostic microbiology laboratories. Public education on preventing infection and reducing transmission. Provider education on diagnosis and management of common infections, antimicrobial use, containment of AMR, disease prevention, infection control. Development, updating and use of essential medicines lists and clinical guidelines. Infection Control Committees to implement infection control programmes in hospitals. Drug and Therapeutics Committees and antimicrobial subcommittees to promote the safe, effective use of antimicrobials. Restriction of availability of antimicrobials. Granting marketing authorisation only to antimicrobials meeting international standards of quality, safety and efficacy. Controlling non-human use of antimicrobials.	2005
Lancet Commission [93]	*”The causes of antibiotic resistance are complex and include human behaviour at many levels of society;* *the consequences affect everybody inthe world. Similarities with climate change are evident”.*	Measuring the extent of the problem and its consequences. Regulation, rational use and infection control in human medicine. The need for rapid diagnostics. The animal sector and the environment. New antibiotics and alternative strategies. Governmental coordination and action. Global governance.	2013
Transatlantic Taskforce [76]	*”AMR is not a new phenomenon; however, the current magnitude of the problem and the speed with whichnew resistance phenotypes haveemerged elevates the public health significance of this issue”.*	Antimicrobial stewardship in human medicine Surveillance of antimicrobial use in human and veterinary communities Risk analysis on foodborne AMR. Campaigns to promote appropriate use in human medicine. Surveillance of drug-resistant infections. Prevention strategies. Incentives to stimulate development of new antibacterial drugs in human medicine. Research to support the development of new antibacterial drugs. Regulatory approaches for antibacterial products.	2014
WHO Global Action Plan [58]	*”Antimicrobial resistance threatens the very core of modern medicine and* *the sustainability of an effective, global public health response to the enduring threat from infectious* *diseases”.*	Improving awareness and understanding of antimicrobial resistance through effective communication, education and training. Strengthening the knowledge and evidence base through surveillance and research. Reducing the incidence of infection through effective sanitation, hygiene and infection prevention measures. Optimizing the use of antimicrobial medicines in human and animal health. Developing the economic case for sustainable investment that takes account of the needs of all countries and increase investment in new medicines, diagnostic tools, vaccines and other interventions.	2015
Swedish National Strategy [97]	*“Antibiotic resistance is a global fate issue that affects us all”.*	Increasing knowledge through enhanced monitoring. Continuing strong preventive measures Responsible use of antibiotics. Increased knowledge to prevent and fight bacterial infections and antibiotic resistance with new methods. Increased knowledge in the community about antibiotic resistance and countermeasures. Supporting structures and systems. Leadership within the EU and international cooperation.	2016
ReAct [60]	*”Antibiotic resistance must be included in the work on sustainable development, and should be seen as a strong additional reason to urgently increase the work on the Sustainable Development Goals”.*	Integrating antibiotic resistance into the development agenda. Developing indicators. Strengthening governance. Stepping up awareness-raising. Implementing interventions with known effect.	2018
The UN Interagency Coordination Group [62]	*”Antimicrobial resistance is a global crisis that threatens a century of progress in health and achievement of the Sustainable Development Goals”.*	Accelerating progress in countries. Innovating to secure the future. Collaborating for more effective action. Investing for a sustainable response. Strengthening accountability and global governance.	2019

Surveillance of resistance and use often appear as a knowledge basis for further development of national agendas. Prevention is linked rather to infections than resistance. Yet the many global efforts need coordination, especially in finding new business models and incentives to stimulate the development of new antibiotics and alternatives [9,100,101].

While strengthening of regulations is urged, an effective, transparent and accountable administrative system is called for. Research priorities are not very visible in the policy recommendations but the need to improve diagnostics and laboratory facilities are.

A recent policy brief from OECD, the Organization for Economic Cooperation and Development [102], sends a strong message to the policy-makers regarding how to ‘Stem the Superbug Tide’. The message is that AMR is a threat to people´s health and without prompt action, 2.4 million premature deaths could occur in Europe, North America and Australasia between 2015 and 2050 as a consequence of AMR. Investing 2 USD per person per year in a package of public health measures could be cost-effective, avoid 47,000 deaths per year in OECD countries and result in net savings of 3 USD per person per year [102].

The most recent report from an international organization was released by the World Bank Group in October 2019. The title “Pulling Together to Beat Superbugs” illustrates well the ever more alarming messages that we are now hearing [52]. The group suggested not only that more but also different types of research are needed in order to identify “knowledge and implementation gaps in addressing AMR.” Specifically, the report calls for moving beyond the laboratory to the community by establishing what works in different contexts through implementation research. The report also confirms the observations made here in this report (Figure 1) that the AMR literature has grown exponentially during recent decades reaching more than 11,000 articles in 2018. The report identifies 23 gaps in four categories; referring to the need to change our current state of thinking reframing AMR as a global development issue, promoting context-specific research environments (enabling) that address how to reduce the need for antibiotics by preventing infections (adaptation) and also how to limit the use of antibiotics (mitigation).

## Health care practices and guidelines

Implementing best practice and guidelines requires dialogue and communication between stakeholders. In order to achieve behavioural change in antibiotic use, we need to convince the public of the negative effects on individuals resulting from unnecessary use. In order to maintain public awareness, continuous communication efforts are essential, e.g. via national and local media. For prescribers, easily accessible summaries of guidelines for common infections have been well received and used [47].

Hand hygiene is acknowledged as a key behavioural determinant, among other measures, for the prevention of infections [103]. Physicians´ perception and motivation to prevent ABR in hospitalized children has been assessed in research using the Health Belief Model. Awareness of the problem was good, although the participating physicians believed that ABR was more of a problem nationally than in their own institution or practice [104–106].

Many of the antibiotics prescribed in primary care are for acute respiratory infections (ARI). However, there is no strong evidence-base for this practice [107]. A study of English primary care found that most prescriptions for antibiotics were meant to treat respiratory and urinary infections, and one third of these were without clinical justification [108]. A study that compared actual prescription rates with the “gold standard” based on guidelines, found that antibiotics were prescribed in 41% of all acute cough consultations. This compared with 10% recommended in expert guidelines [109]. There are major variations in prescription rates between primary care practices. Yet, knowledge in this area is lacking, in part because of differences in morbidity and/or health seeking behaviours [110].

A systematic review and meta-analysis of 24 studies (including 19 observational) concerned with prescribing in primary care, reported pooled odds ratios of 2.5 and 2.4 respectively for urinary tract and respiratory tract bacteria resistance within two months. This implies that bacterial resistance to antibiotics prescribed for a respiratory or urinary infection in primary care are more than twice as likely than elsewhere. The effects, which were strongest in the first month after treatment, lasted for a year [111].

The process when a healthy person develops symptoms of ARI and consults a general practitioner, who may or may not prescribe antibiotics, has also been analysed [107]. Studies suggest that communication and shared decision-making between the patient and the physician can reduce prescriptions for ARI. Patient satisfaction is associated with information, not only with antibiotic prescriptions. Misuse often goes with diagnostic uncertainty and delayed prescription can be a preferred option. This strategy has also been shown to reduce antibiotic use compared with that of giving immediate prescriptions [112].

An evaluation of the impact of the French guidelines on antibiotic prescriptions for acute otitis media in infants showed that the prescription of broad-spectrum antibiotics decreased significantly in both private practices and in pediatric emergency departments [22], although an audit of outpatient prescriptions showed that only 40% were consistent with the guidelines [113].

A Swedish example of poor compliance with national guidelines was recently reported from ophthalmology where, in a retrospective cohort study, antibiotics were prescribed for more than two thirds of the diagnosed 32,000 cases of conjunctivitis, which is often a self-limiting condition [114].

Addressing ABR in intensive care units has been a priority because these settings are breeding and spreading antibiotic-resistant pathogens [115]. Strict adherence to clinical guidelines based on culture data, optimal dosing and duration are critical [116]. Cyclic administration between different antibiotic classes has been advocated for intensive care units although this is still subject to limited efficacy. We also lack conclusive evidence regarding whether combination therapy reduces ABR [117,118].

Self-medication is a major contributor to ABR. This is especially common in socio-economically deprived societies where there is poor knowledge and awareness among the public, and critically, among many care givers [20]. A review of 34 studies from LMICs with 31,340 participants, reported the overall prevalence of self-medication as 39% [119]. Most common malpractices were too short treatment duration, insufficient dose, use of wrong drug and inappropriate sharing of medicines.

The authors of a systematic review which assessed public knowledge and beliefs about ABR screened 3,537 articles published in Europe, Asia, and North America covering 55,225 persons [120]. Both quantitative and qualitative data were assessed. About 70% of participants had heard about ABR in the past, 88% believed that it referred to some sort of physical change in the body, while the rest believed that the excessive use of antibiotics, rather than failure to complete antibiotic courses, led to the resistance problem. As many as 84% suggested that ABR could be reduced by limiting the use of antibiotics and seeking help from clinicians. The qualitative data showed that people considered the emerging ABR crisis as a consequence of other people’s actions and that clinicians should find a remedy. The researchers concluded that the public is not fully aware of what exactly the ABR crisis entails and have incomplete knowledge about antibiotic use [120].

While ARI is a leading cause of death among under-five children in low-income countries, irrational use of antibiotics worsens the situation. A community intervention in rural Vietnam targeted health care providers through education, case scenario discussions and poster campaigns. A mixed-methods evaluation showed significantly improved knowledge about ARI and antibiotic practice in the intervention compared to the control group [121].

Travel medicine has been called upon as a key player in preventing the spread of ABR [122] since travellers are vectors in transporting bacteria across regions. Out of the 300 million visitors to poor hygiene countries each year, 60–150 million are estimated to return with diarrhoea, that is wrongly self-treated with antibiotics, which do not prevent them from becoming carriers of resistant bacteria, which in turn may be transferred to others.

The overall use of antibiotics has decreased in Sweden over the past 20 years without notable negative consequences. Levels of ABR are relatively lower than in other countries. In a report on lessons learnt, progress is attributed to committed work among different professions. Continued strong political support is deemed essential. A key element for achieving long-term change has been the bottom-up approach. This involves working closely with prescribers at the local level. Future work will build upon this to achieve a sustainable approach to controlling ABR [47].

## Preventive strategies

The major clinical strategies for ABR prevention are infection control, infection-specific prevention protocols, and antibiotic management strategies [123]. In a systematic review, summarising progress in preventative techniques [2], the authors asked the rhetoric question ‘why are humans not better equipped to face the microbial world’. Their prompt response was that “microbes have been evolving far longer than humans … that bacteria can replicate their entire genome in 20–30 minutes, a task that takes humans 20–30 years to complete. Thus, microbes can arguably qualify as the most plentiful, diverse and adaptable species on the planet”.

Any global health policy aimed at reversing ABR has to consider the needs to ensure universal access to essential medicines. Weighing the associated risks against limited access is difficult. Failure to balance excess and access is an ethical neglect [15,124]. A Lancet Commission called for a global code of conduct as part of implementing preventive strategies for ABR [93]. One has to accept that self-prescription may be the only way for some people to gain access to medical services and banning all non-prescriptions may not be a solution.

Four ethical challenges have been identified – the conflict between patient autonomy and the protection of others, the ability of health systems to distribute resources fairly, the impact of animal welfare complying with restricted access, and the preserving of a limited public good across generations. A study of 400 general physicians and 429 infectious disease doctors addressed the tension between societal concerns and individual patients. These clinicians were more motivated by the idea of treating the individual patient than protecting society [124]. Doctors´ethical dilemmas of balancing the autonomy of current against future patients may be a challenge to the trust between the doctor and the patient. These are all issues that need attention by researchers as well as policy makers with due account of different national contexts [125]. It is also important to better understand how organizational structures and processes impact on the implementation of preventive strategies and policies [126].

It has been suggested that in order to counteract antibiotic resistance we need to break the barriers among previous antibacterial strategies. The concept of ‘one infection, one bug, one drug’ no longer applies. Resistance has to be attacked multilaterally in patients who are sick because of resistant infections, in groups of patients and healthy people harboring resistant microbes, and in the environment where resistance develops. Prevention strategies therefore need to acknowledge this complexity and move beyond treating the individual only [127].

An overview of preventive strategies for Australian hospital care, where 200,000 health care acquired infections occur annually and 50% are preventable, presented a long list of strategies, some specific for each specialty, and others generic, such as hand hygiene. The authors noted that the success of such strategies will require a cultural shift with institutional as well as individual accountability and adherence to guidelines [128].

Advertising is a common strategy used by the pharmaceutical industry in marketing their products. In a study analysing 779 advertisements in 24 medical journals, antibiotics was addressed in 25% of these. None, however, mentioned antibiotic resistance or the need to prevent irrational use of antibiotics [129].

It is generally acknowledged that the agenda for combating antibiotic resistance differs between high- and low-income countries. More children die from a shortage of antibiotics in low-income countries than from drug-resistant infections. Efforts therefore have to balance access and misuse [18]. In particular, such a strategy must not only focus on human health but also consider the growing impact on resistance from animal and environmental exposures [130]. It is estimated that countries that consume most veterinary antibiotics are all LMICs. Furthermore, not only is population growth biggest in those countries, but economic development, while fostering general health development, is expected to occur in parallel with increased animal-based protein consumption.

## What do people know?

Numerous epidemiological studies on ABR have been undertaken in high- middle- and low-income countries. Most of these studies have been descriptive using cross-sectional surveys addressing questions such as awareness, knowledge, attitudes and practice.

A study across 12 countries (Nigeria, South Africa, Barbados, Mexico, India, Indonesia, Russia, Serbia, Egypt, Sudan, China and Vietnam) was conducted in 2015 as part of the implementation of objective 1 of the WHO Global Action Plan ‘to improve the awareness and understanding of AMR through effective communication, education and training’. The study, which included 9,772 respondents, found that 65% had taken antibiotics during the prior 12 months, and 35% within the last month. The latter figure was 42% in low-income countries and 24% in people aged 65 years and older. Twenty-five percent of people across the 12 countries thought that it was acceptable to use antibiotics left-over from others (if prescribed for the same illness) and 32% reported that they generally stop taking antibiotics when they feel better. Two-thirds of the respondents believed that colds and flu can be cured with antibiotics. Some correct actions, such as regular hand-washing (91%) and only taking antibiotics when prescribed (87%) were acknowledged. The study revealed major misunderstandings regarding the meaning of ABR. While there was high awareness about increasing ABR (72%), three quarters incorrectly believed that their body, rather than bacteria, become resistant to antibiotics. Many also held the view that it is only a problem for those who take antibiotics regularly [131].

Population-level knowledge was investigated in a systematic review and meta-analysis of 24 studies, nine from Europe, ten from Asia, four from North America and two from Oceania. The findings showed that half the sample did not know that antibiotics were ineffective against viruses. Although 59% were aware of ABR, 27% did not know that misuse could lead to resistance. Through a meta-analysis it was estimated that more than 50% of the sample revealed inappropriate behaviour regarding antibiotic consumption [132]. In looking at the misuse of antibiotic therapy, another systematic review and meta-analysis found that one third of patients did not comply with prescriptions in terms of dosage and duration, and one quarter kept left-over antibiotics for future use [133].

Contrary to what one would expect, there is no consistent association between general education and antibiotics knowledge. There is evidence that personality traits like neuroticism, agreeableness and conscientiousness are associated with adherence to antibiotic prescriptions [134]. Achieving behaviour change is often more challenging than just conveying knowledge.

In a study conducted in Macedonia, more than 60% of participants said that they knew that antibiotics were effective against bacteria, but only one in four understood that they were not effective against viruses. Less than one in ten knew that antibiotics do not work on most coughs and colds. Sixty-eight percent of the participants reported that they were prescribed antibiotics by their doctors during the year prior to the survey. In the same period, 72% reported that they had stored antibiotics at home, and 43% reported that they had purchased antibiotics without prescription [135].

In a study of secondary school students in India, two thirds were unaware of the problem of ABR and almost half thought that bacteria caused colds or influenza. Around one in five said that they frequently self-medicated with antibiotics, more than half reported stopping antibiotics when symptoms were gone, and one in four said that they stored unused antibiotics at home [136].

A qualitative study, also from India, explored community perceptions of ABR. The participants perceived an increased climate variability with health consequences for their community. They acknowledged an interrelationship between the environment, infectious diseases and antibiotic use although their understanding of ABR was poor. Quantitative data substantiating the relationship between climate, infectious diseases and drugs are basically lacking [137].

The perceptions of antibiotic access and use have recently been studied within a Health and Demographic Surveillance Site in rural South Africa. Qualitative interviews and focus groups were held with 60 community members. They suggested the need for strong sales regulations and context-specific educational campaigns to promote the safer use of antibiotics [138].

A study from Jordan revealed parental misconceptions about antibiotic use in children with upper respiratory infections which two thirds ascribed to weather change. Many said that they would give antibiotics without prescription when the child had a sore throat, ear pain, fever, cough or common cold [139].

A study from Kuwait issued a self-administered questionnaire to a random sample of 770 individuals. Three quarters reported that they had been prescribed antibiotics during the previous 12 months, one in three of whom did not finish their course. Self-medication was common and there was poor general knowledge about use, safety and resistance [140].

A recent German study showed that people´s knowledge of antibiotics depends on their previous experience of having taken antibiotics. This suggests that health literacy, the ‘ability to understand and critically evaluate health information and to make health-related decisions’, may be part of the mechanism to promote responsible use of antibiotics. In a multivariate analysis, individuals with sufficient health literacy, operationalized on a 16-point scale instrument, were almost half as likely to have used antibiotics recently, compared with individuals with insufficient health literacy [141].

The Swedish population has fairly good knowledge and awareness about the use of antibiotics and the implications of resistance. In an interview study of 1,000 individuals, aged 21 to 80 years, 19% thought that antibiotics could cure common colds. This is considerably lower than previously reported from the UK and US. High trust was expressed in doctors who did not prescribe antibiotics [142].

In a European survey conducted in 2013, Swedish participants were among those with the highest overall knowledge of ABR among the 27 participating countries. Among the 1035 Swedish participants, 98% agreed with the statement ‘unnecessary use of antibiotics make them become ineffective.’ Half of the Swedish respondents recalled receiving information on ‘not taking antibiotics unnecessarily e.g. in the case of cold or flu’ in the last 12 months. Seventy-seven percent knew about antibiotic ineffectiveness against viruses and the common cold compared with the EU average of 52%. Although knowledge of ABR in the Swedish population has increased, there is still room for improvement, especially among the lower educated and among the elderly [143].

In a study of Swedish travellers, they were mostly aware of what the term ‘antibiotic resistance’ meant, but did not understand its real significance, or the consequences for the individual or for society. Few thought that they might be carriers of resistant bacteria. Many travellers believed that ABR was only a problem in developing countries and that it did not affect healthy people. They did not feel personal responsibility for the development of ABR [144]. Travel medicine practitioners have a responsibility as gatekeepers for appropriate prescribing of antibiotics for the common condition known as ‘travellers diarrhoea’ [122].

It is a common notion that people do not see the association between their personal use of antibiotics and the global picture of ABR, a pattern resembling the disconnect between personal behaviour and knowledge about global warming. This was addressed in a combined mixed methods study in Hong Kong in which focus groups were used to develop a survey questionnaire instrument. The responses indicated that people believed that ABR was a distant non-personal threat for which doctors were responsible [145].

People who have less trust in the health care system are less likely to comply with prescriptions [146]. It is also acknowledged that delayed initiation of treatment awaiting culture is medically motivated most of the time. In order to test the so called ‘collective action dilemma’ which means that, in order to balance individuals´ short-term interest in antibiotic treatment collective support may be needed, a study was conducted in which respondents in different scenarios were asked how long they could accept postponing antibiotic treatment. The answers correlated positively with the number of days they were told that others were willing to wait, indicating that the generalized trust and proclaimed public willingness influenced the individual´s willingness [146].

The authors of a systematic review of 54 quantitative and qualitative studies of public knowledge and beliefs about ABR concluded that the public often has incomplete understanding of, and misperceptions about, ABR. Many participants also believed that they do not contribute to the development of resistance and that they themselves are at low risk. Their conception of ABR was that of a change in the body rather than in the bacteria. The synthesizing of the results of both qualitative and quantitative studies provides further understanding of knowledge and beliefs about antibiotic resistance [120].

## What do prescribers know?

Prescribing at best occurs through a consultative dialogue between the patient and the doctor. Knowledge and expectations for both come into play. Doctors are expected to be well-informed. However, in a systematic review of 57 published studies, which included almost twelve thousand clinicians, most of which from North America and Europe, 89% recognized ABR as a global problem but only 67% saw it as an issue of relevance for their own practices. In qualitative interviews, doctors acknowledged that ABR was a serious problem, but one caused by others. They saw ABR as a big problem in their own countries, but being of less importance in the hospitals where they work, and not of particular relevance on their clinics [77,147].

As part of the implementation of a US campaign to prevent ABR, 695 clinicians took part in surveys, in-depth interviews and focus groups to assess barriers to prevent AMR in healthcare settings [147]. When evaluating the campaign´s four main strategies they ranked ‘Diagnose and treat infection effectively’ and ‘Use antimicrobials wisely’ higher than ‘Prevent infection’ and ‘Prevent transmission’ [147].

Attitudes and diagnostic uncertainty are key drivers of drug use and misuse. Fear of being sued for not prescribing at all, or prescribing the wrong antibiotic, is the reality in many countries. Yet guidelines are perceived by some as a challenge to the clinician’s autonomy or as rigid ‘cookbooks’ [33]. When European medical students were asked to share their perceptions about antibiotics, they requested more education to improve their confidence regarding prescribing decisions [148]. Similar results were found for Ethiopian paramedical students who, although they thought ABR was preventable, had poor knowledge about causes and consequences [149]. In a survey of knowledge, attitudes and antibiotic prescribing among 800 Jamaican physicians 82% regarded ABR of global importance, 73% of importance nationally and 53% of importance in their personal practices. Only 7% reported that they always took cultures before prescribing antibiotics [150].

A multi-country qualitative study conducted across seven LMICs, explored ABR awareness among 244 human and animal healthcare professionals. Although awareness was high, this did not translate to lower prescribing [151]. The participants reported that antibiotics were prescribed as hygiene replacement when facilities for sanitation were lacking or as a ‘safety measure’ in malnourished children, even when the infection was not shown to be bacterial. The findings suggest that awareness is insufficient to change behaviour without taking into account cultural norms, economic and health infrastructures. This multi-country study conveys the important theoretical and methodological message that there is no causally linear relationship between the K (knowledge), A (attitudes) and P (practice) in the numerous KAP surveys. The study also challenges the common notion of ‘blaming the victim’ for poor knowledge when, instead, resources are lacking for appropriate daily health care [151].

In a qualitative interview study with doctors, veterinarians and drug dispensers in East India, respondents requested more information about the inter-relationships between antibiotic use, resistance development and the environment, as well as the enforcement of legislation [152]. In a study in Syria, where antibiotics are commonly dispensed through pharmacists without prescriptions, most respondents did not think that ABR posed any problems for patients [153]. This calls for the need to ensure responsibility and better communication between pharmacists and patients.

A study from Ghana in which most prescribers were nurses, showed that knowledge about ABR was high, although there were gaps in the knowledge and perception among different prescriber categories. The interviewees requested more formal information about ABR to support prescriber’s antibiotic prescription practices [154].

On behalf of the Swedish Government, the Swedish Public Health Agency commissioned two behavioural studies to analyse factors which determine general practitioners´ prescriptions for antibiotics [155]. A combined quantitative-qualitative study looked at respiratory problems at Swedish health care centres. Low-prescribing centers were characterized by inter-professional cooperation across different work specialties and a critical role for nurses. Access to, and feedback of data on one’s own prescriptions was prioritized. Interviews with 25 physicians assessed compliance with guidelines. Those who did not comply often had outdated knowledge and disagreed with their patients. The Agency concluded that a more systematic approach to research-based quality development should be initiated in primary care organizations and that this should focus on implementing established guidelines.

## Alternatives to antibiotics

There is no known single universal future pathway that provides a remedy for ABR. It is probably safe to say that we need to use the antibiotics we have more wisely while developing new products in parallel. This report mainly deals with the former strategy. Introducing new antibiotics would seem to be the most obvious strategy but a look back at the fate of previous classes of antibiotics does not provide much hope. Nor does the pharmaceutical companies´ pace in product development. New business models in public-private partnerships may stimulate drug development but, at the same time, alternative prevention as well as treatment strategies must be developed [9].

Whether vaccination is a realistic and warranted strategy remains a challenging question [156]. What populations should be targeted, will immune-suppressed individuals respond, and what are the economic implications? Another strategy, although still at the modelling stage, suggests ‘fight evolution with evolution’ [157]. This refers to steering evolution with drug sequencing through the genotype space to states from where resistance cannot emerge [158].

In a Lancet Review, published in 2016, commissioned by the Wellcome Trust, 24 scientists identified alternatives to antibiotics. Of those that could be used in combination, 10 were analysed in more detail. In terms of high clinical impact and high technical feasibility, the approaches which were expected to have the greatest potential to provide alternatives to antibiotics were: phage lysins as therapeutics (viruses that can kill bacteria via their enzymes, lysins); vaccines as prophylactics; antibodies as prophylactics (restricting the ability of bacteria to cause illness), and probiotics (preventing bacteria from colonizing in the gut).

Although not unanimous in their views, the Lancet authors concluded that we must rely on alternatives to antibiotics in the future. They suggested that a major international science programme, funded for 10 years, would be required to provide the solutions that are urgently needed [159].

As reflected by the literature search conducted as part of this scoping review (Figure 3), several papers on the preventive potential of probiotics have been evaluated as alternatives or complementary therapies among the 399 papers included in this review. This has motivated a brief summary of them in this section of the report.

Probiotics may serve to reduce symptoms, act as treatment alternatives for milder infections and reduce the likelihood of prescribing antibiotics. Probiotics are defined by FAO and WHO as ‘live organisms which, when administered in adequate amounts, confer a health benefit on the host’. While probiotics and food supplements have gained public and commercial popularity, academic interest has not followed and research in these areas has been lagging behind [160]. Single studies provide limited stand-alone evidence. Hence, it is important to undertake reviews in which data are pooled and results summarized, accounting for bacterial strains.

A comprehensive overview of probiotics looked at their potential role in treating and relieving symptoms for a number of diseases and specifically whether probiotics could substitute for antibiotics in certain infectious diseases. This body of work which was completed as an independent project in Biology [161] showed that probiotics can stabilize the intestinal microbiota (microflora), disturbances of which, apart from the resistance problem, is one of the key side-effects of antibiotics. One of the most obvious positive things about probiotics is therefore their ability to prevent antibiotic-associated diarrhoea (AAD), e.g. by co-administration of probiotics during antibiotic treatment. Several studies and reviews have also addressed this issue.

In view of the increased recognition of the microbiota as a target for infection prevention and control, epidemiological studies are called for to investigate how probiotics impact on, and potentially promote, biodiversity [162]. Caution has however been expressed. If used as an alternative to antibiotics, probiotics may transfer antibiotic-resistant genes from the intestine to other sites [163]. More research is therefore needed to assess the efficacy and potential side effects of probiotics as a means of reducing the emergence of resistance.

In a systematic review of 17 RCTs, the authors assessed whether probiotic consumption would reduce antibiotic consumption for common acute infections among children. The findings showed that infants and children who received probiotics had a 29% lower risk of being prescribed antibiotics than a placebo group [164].

After reviewing 12 studies assessing the ability of probiotics to prevent recurrent UTI in women, the authors concluded that there is low to moderate evidence favouring the effectiveness of probiotics and that larger randomized and double-blind studies are needed to strengthen recommendations [165]. In view of the increasing consumption of probiotics there is also a need for proper safety studies and evidence-based documentation [166].

Of late there have been suggestions that probiotics can safely impact on a number of physiological functions and enhance the immune system [167]. A meta-analysis of 25 RCTs with 2810 AAD patients, estimated that probiotics reduced diarrhoea by 57% [168]. Exploring the role of probiotics in reducing resistance spread, the CDC concluded that ‘the role of probiotics in preventing drug-resistant infections in humans has not yet been established and although some studies have shown benefit, the data is currently not conclusive enough to issue specific recommendations.’ Thus, so far probiotics can be used as partial replacement or adjunct to antibiotic treatment. Hence in this way they can possibly be of benefit in treating multidrug resistant UTIs [168].

A Cochrane review of paediatric cases, which included 23 studies and 3938 participants, showed an overall incidence of AAD of 8% in the probiotic group, compared with 19% in the control group, i.e. a protective effect of 54%. The study also suggested that more safety data are needed to determine dosing and types of microbes for probiotics [169].

The above results are similar to results from a systematic review and meta-analysis of 20 trials with 3818 participants, both adults and children. The findings showed an overall reduced risk of diarrhoea of 66% (78% in adults and 60% in children) indicating that probiotic prophylaxis would prevent 33 episodes of diarrhoea per 1,000 persons. In the probiotic group 9.3% experienced adverse events compared with 12.6% in the antibiotic control group [170].

Analysis of 23 trials examining C. difficile-associated diarrhoea (CDAD) cases, involving 4213 adults and children, showed that probiotics, taken in conjunction with antibiotics, reduced the risk of CDAD by 64%. The incidence of CDAD was 2.0 percent in the probiotic group compared with 5.5 percent in the placebo, or no-treatment control group. Probiotics reduced the incidence of diarrhoea in patients with C. difficile, but did not appear to reduce the incidence of C. difficile infection. In the short term, taking probiotics in conjunction with antibiotics appears to be a safe and effective way of preventing diarrhoea associated with C. difficile infection. However, ‘we still need to establish the probiotic strains and doses that provide the best results, and determine the safety of probiotics in immunocompromised patients.’ [171].

A review of both in vitro and in vivo studies suggested that probiotics have the potential to manage helicobacter pylori infections in paediatric patients [172]. The mechanism by which probiotics interact with the immune system is still unresolved. There is still no convincing evidence to support the use of probiotics as an adjunct to increase the H.pylori eradication rate, mainly because statistical power has so far been insufficient in clinical trials.

However, there appears to be sufficient evidence to justify the use of probiotics as an alternative to antibiotics for some milder infectious diseases [169–171]. The most convincing evidence that an alternative to traditional antibiotic treatment actually works is the use of fecal microbial transplantation (FMT) to treat CDAD [173]. Here, the approach is to transfer a healthy donor microbiota and increase the microbial diversity in the recipient gut and by this compete out the resistant or toxin producing bacteria. Consequently, a well-balanced diverse microbiome may prevent colonization by resistant bacterial clones. Future research must equally address efficacy and potential harm for such alternatives as done for many antimicrobials.

## Conclusions

An overall interpretation from this review is that descriptive rather than theoretical ambitions have characterized the literature. A common denominator in many policy documents is the call for more and better surveillance systems. There is a wealth of information on the local and national uses and misuses of antibiotics. Numerous educational and stewardship programmes have been published. In addition, there are several studies covering the knowledge of the public and prescribers. Epidemiological modelling needs strengthening, especially in LMICs. New tools drawing on social media are needed. If we want to better understand and explain the antibiotic situation from a behavioural perspective, the required approaches are lacking.

The following paragraphs summarise knowledge gaps according to the structure in Figure 3 and a concluding *know-do list* is presented in Table 2.

**Table 2.** A summary of suggested research strands on “prevention of antibiotic resistance”

Research area	Know-do gaps
*Epidemiology and Surveillance*	Explain differences in antibiotic usage and resistance across Europe in longitudinal cross-country studies. Address the global divide between excessive use in well-to-do regions and inadequate access in poor regions. Global surveillance and estimates lack valid data from LMICs. Quantitative assessments need to be supplemented with qualitative and social research. Assess changes in resistance following decreased drug consumption.
*One Health – an interdisciplinary and intersectoral issue*	Bridging is needed between biomedical, environmental, animal and social sciences. Implementation research should address unused knowledge to be put into practice. Closer collaboration and better documentation of antibiotic use across sectors are called for. Address the economic forces behind infectious diseases and antibiotic resistance. Better understanding and documentation of the irreversible consequences from not taking actions are needed. ABR needs to become a high-profile social issue, like cancer, tobacco control and HIV/AIDS.
*Understanding what drives AMR*	Analyse the population impact of different drivers of antibiotic resistance. Assessing the direct effects of antibiotic use on resistance need clinical studies on bacterial culture and colonization. Assess relations between antibiotics prescribed in primary care and infections that require secondary care treatment. Clarify the effects of antibiotic dose, duration and adherence on resistance. For interpretation of intervention studies we need randomized studies with proper control groups.
*Antimicrobial stewardship*	Strengthen the evidence base for programmes in hospital settings through systematic reviews and meta-analyses. Mixed evaluation models are needed supplementing quantitative methods with qualitative approaches. Behavioural science is lacking in the development and evaluation of antimicrobial stewardship programmes. Account should be taken of the cultural and social norms around prescription and clinical practice. Stewardship programmes outside hospital settings, e.g as community or public campaigns have not been assessed. In view of the importance of context, information on stewardship programmes is generally lacking from LMICs. Provide solid and generalizable data on cost and cost-effectiveness of hospital stewardship programmes.
*Antibiotic resistance – a policy and health systems issue*	Strengthen the evidence base for the effectiveness of the numerous policies across the human and animal sectors. Stimulate the development of new antibiotics and evaluation of alternatives. New business models are called for where public stakeholders involve private partners. Prevention of ABR should be more visible in Agenda 2030 and more closely related to its SDGs. Research comparable and in analogy with that regarding other global health threats should be encouraged. Preserving antibiotic effectiveness ensuring universal access, is an ethical obligation warranting in-depth research. Analogies with other global concerns can help us understand scope and consequences of no-action.
*Health Care Practices and Guidelines*	Knowledge is lacking about major variations in prescription rates between primary care practices. Cyclic administration of antibiotics has been advocated for intensive care but is still subject to limited efficacy. We lack complete evidence that combination therapy reduces ABR. Research on ABR prevention needs to move beyond the laboratory to the community.
*Preventive Strategies*	We need to better understand how organizational structures and processes impact on preventive strategies and policy. Address the tension between societal concerns and individual patients. Advertisements in medical journals are an interesting venue for research on ABR communication.
*What do people know?*	How do people perceive of ABR in comparison with other global threats? How do people in different ages and cultures cope with antibiotic resistance? How do people look upon the responsibilities for change for the individual versus society? KAP surveys on ABR need improvement through stronger theoretical frameworks.
*What do prescribers know?*	Communication research on the dialogue between the patient and the doctor should be stimulated. Synthesis of qualitative and quantitative studies may enable more in-depth explanations of knowledge and beliefs. Interventions are called for to improve prescribing behavior and rebuild patient-physician trust. Qualitative research could address how social norms and standards of care can influence prescribing behaviour.
*Alternatives to antibiotics*	Establish the probiotic strains and doses that provide the best results and safety in immunocompromised patients. Assess efficacy and potential side effects of probiotics as a means of reducing the emergence of resistance. Assess evidence favouring the effectiveness of probiotics through larger, randomized and double-blind studies. Undertake proper safety studies including documentation of dosing and types of microbes.

*Epidemiological* longitudinal studies are called for to explain the wide differences in ABR and antibiotic usage across Europe. The fact that the global divide is characterised by excessive use in well-to-do regions and inadequate access to antibiotics in poor regions, needs stronger research recognition. Global *surveillance* mainly suffers from a lack of valid data from LMICs. The existing health and demographic surveillance sites would enable valid cross-cultural comparisons, if supplemented with data on resistance and antibiotic use. Quantitative assessments need to be supplemented with qualitative and social research to better understand why antibiotic usage varies between cultural settings and societies. Despite a strong theoretical basis for the prediction of resistance development, few epidemiological studies have recorded temporal changes in the frequency of resistance to a specific drug when the volume of drug consumption in the community is intentionally reduced.

The *One-Health* strategy acknowledges the need to bridge the gap between the biomedical, environmental, animal and social sciences. Furthermore, implementation research is needed in which interventions are designed and evaluated using existing knowledge. Closer collaboration and better documentation of antibiotic use across sectors is required, since more antibiotics are used for animals to produce food, promote growth and prevent disease than for humans. The economic forces behind infectious diseases and resistance development need to be addressed. In order to better understand the irreversible consequences which will result from not taking action, contextual and cultural determinants must be tackled globally. The ABR concern needs to become a high-profile social issue, like cancer, tobacco control and HIV/AIDS. ABR must be perceived as being of relevance to the lives of each and everyone across different settings and cultural contexts.

In order to design preventive intervention studies, we need to better understand the population *impact of different drivers* of ABR. Epidemiological studies assessing the effects of antibiotic use on resistance will also need clinical studies and data on bacterial culture and colonization. Research is also needed to assess relationships between antibiotics prescribed in primary care and more serious infections that require secondary care treatment, as well as to further clarify the effects of dose, duration, and adherence in antibiotic treatment on the development of resistance. Rigorous randomized studies with proper control groups are needed.

Although a number of evaluations have been performed in hospital settings, the evidence base for *stewardship programmes* is weak since program diversity and suboptimal study designs have made systematic reviews and meta-analyses less valid. Evaluation models supplementing quantitative methods with qualitative approaches are needed. Account should be taken of the cultural and social norms around prescription and clinical practice. Programmes outside hospital settings, e.g. community or public campaigns, have not been assessed. Context is important and more studies are needed in LMICs. Rigorous generalizable data on cost and cost-effectiveness of hospital stewardship programs are needed.

The mere absence of progress in combating ABR is partly due to a poor evidence base for the effectiveness of the numerous *policies* across the human health and animal sectors in both HICs and LMICs. In order to stimulate the development of new antibiotics and the evaluation of alternatives, new business models are called for where public stakeholders may involve private partners. In policy analyses, the prevention of ABR should be more visible in Agenda 2030 and integral to the SDGs. Analogies with other fundamental global concerns, such as climate change, can help us to understand the scope and irreversible consequences we face if decisive and far-reaching actions are not taken. Preserving antibiotic effectiveness while ensuring universal access is an ethical obligation and concern warranting in-depth and multidisciplinary research. ABR raises a number of ethical issues for research, e.g. individual liberty versus public health, global access versus overuse of antibiotics, intergenerational fairness, and our collective responsibility for the wellbeing of future generations.

Implementing best *health practice and guidelines* requires dialogue and shared decision-making between the patient and the physician. Misuse often goes with diagnostic uncertainty and delayed prescription can be a preferred option but we lack evidence on the efficacy of e.g antibiotic cycling and combination therapy. We need also to convince the public of the negative effects on individuals resulting from unnecessary use. For prescribers, easily accessible summaries of guidelines for common infections have been well received and used. From lessons learnt in Sweden over the past 20 years, progress is attributed to committed work among different professions. A key element has been the bottom-up approach working closely with prescribers at the local level.

It is important to better understand how organizational structures and processes impact on the implementation of *preventive strategies* and policy. There are major variations in prescription rates between primary care practices but it is not known to what extent this is due to differences in morbidity and/or in health seeking behaviours. Research on ABR prevention needs to move beyond the laboratory to the community by establishing what works in different contexts through implementation research. The tension between societal concerns and individual patients are all issues that need attention by researchers as well as policy makers. Advertisements in medical journals may be a potentially interesting venue to open communication channels about ABR research among clinicians.

Surveys on knowledge, attitudes and practices need stronger theoretical frameworks since assumptions of causal linear links between components are often spurious. There are a number of questions to be addressed. How do *people* perceive ABR in comparison with other global threats such as terrorism or climate change? How do people in different ages and cultures cope with ABR? What do they expect from health care and health policy? How do they look upon the responsibilities for change in regard to the individual versus society?

*Prescribing* is an important communication conduit between the patient and the doctor, and as such, is worthy of increased interest from researchers. The synthesis of the findings of qualitative and quantitative studies may enable more in-depth explanations of knowledge and beliefs about ABR than would have otherwise been achieved using only quantitative data. Interventions are urgently needed to promote appropriate antibiotic prescribing by doctors, enforce restrictions on the sale of over-the-counter antibiotics without prescriptions, and educate the general public about the management of self-limiting illnesses. Interventions should improve prescribing behaviour and rebuild patient-physician trust and understanding when antibiotics are not prescribed. Doctors´ethical dilemmas of balancing the autonomy of current against future patients may challenge that trust. Qualitative research is needed to better understand how the social norms and standards of care that influence antibiotic prescribing behaviour can be changed.

The wiser use of existing antibiotics needs to be matched with efforts to develop new antibiotics and with the development and evaluation of *alternatives*. Research must address their efficacy and potential harm as has been the case for many antimicrobials. More research is needed to assess the efficacy and potential side effects of probiotics and fecal donation (FMT) approaches as a means of reducing the emergence of resistance. To assess evidence favouring the effectiveness of probiotics and FMT we need larger, randomized and double-blind studies to strengthen recommendations.

## Epilogue

**Figure 5.**
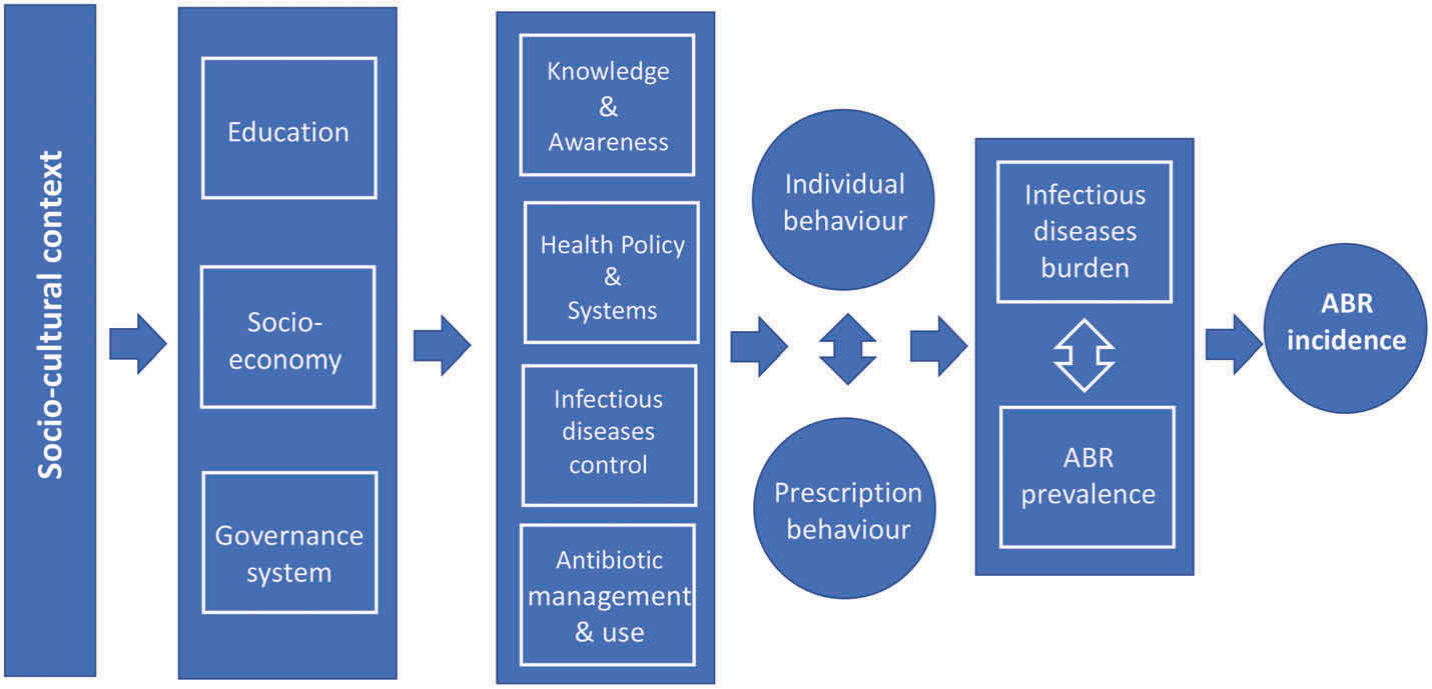
A suggested epidemiological framework with potential entry-points for prevention of ABR

This report has followed the thematic structure displayed in Figure 3 and which emerged during the process of screening for relevance an initial number of 622 references by titles and abstracts and in the final reading of 399 complete articles. Figure 5 is an attempt to synthesise the findings in an epidemiological framework and causal web. The figure illustrates the presumed causal web from background and intermediate factors towards the behavioural interplay between individuals and prescribers ultimately affecting ABR occurrence as modified by a setting´s burden of infectious diseases and prevailing ABR.

Any intervention is context-specific and the literature convincingly claims that the socio-cultural setting is an ultimate background factor and determinant. Especially, conditions and opportunities differ between low-, middle- and high-income countries, as measured by different education levels, socio-economic situations and governance systems. These affect their populations´ knowledge and awareness of ABR, their health policy and systems´ priorities and commitments to infectious disease control and which is manifested in guidelines for antibiotic management and use also beyond the health sector. Individuals´ and prescribers´ behaviour, that are interdependent, constitute intermediate outcome variables. Their effects on ABR are modified by the infectious diseases burden of the actual setting in pushing current ABR prevalence towards additional or prevented incident cases of ABR.

The framework may potentially serve to identify entry points for the prevention of ABR. It also suggests a needed focus on population-based and inter-disciplinary research and community interventions complementary to the current well-established biomedical and clinical approaches to tackle ABR. Such a broadened orientation should preferably also be reflected in the funding landscape.

The work for this report began two years ago with a database search for published literature on the ‘prevention of antibiotic resistance’. An impressive amount of interest has been devoted to the topic of ‘antibiotic or antimicrobial resistance’ during the past two decades. However, the ‘prevention of resistance’ has attracted less interest than the ‘prevention of infections’. Yet, interest is increasing and ABR is now acknowledged by many as being of critical importance nationally as well as globally. The lessons for policy are evident in the many alarming reports from organizations referred to in this overview. The recognition of ABR as a global threat and the need to act collectively is appropriately urged by Tedros Adhanom Ghebreyesus, WHO´s Director-General: *‘Working together is the only way to avoid the huge human, social, economic, and environmental costs of antimicrobial resistance’* [174].

## Paper context

Antibiotic resistance is a threat to global security alongside terrorism and climate change. This review documents increasingly alarming policy reports while stronger academic responses on evidence based preventive strategies are called for. Specifically, valid surveillance systems in low- and middle-income countries are lacking. The review also suggests a needed focus on population-based and inter-disciplinary research complementary to the current well-established biomedical and clinical approaches to tackle ABR.
